# 27 years experience with the gastroepilopic artery graft in CABG

**DOI:** 10.1186/1749-8090-8-S1-O206

**Published:** 2013-09-11

**Authors:** H Suma

**Affiliations:** 1 Suma Heart Clinic, Tokyo, Japan

## 

To improve the long-term outcome after CABG, several strategies have been used using arterial conduits.In 27 years experience with the right gastroepiploic artery (GEA) graft, 1552 patients having CABG with the GEA graft, (1142 men, mean 64 years, 99% multivessel disease, and mean EF 0.51), internal thoracic artery, saphenous vein, and radial artery grafts were concomitantly used in 1505 (97%), 853 (55%), and 130 (9%) patients, respectively. The mean number of distal anastomoses was 3.2, and 2.4 coronary arteries were bypassed with arterial grafts. The sites for GEA grafting were 70 anterior descending, 318 circumflex, and 1239 right coronary arteries. The operative mortality was 1.26%. In 1118 follow-up patients (72.0%), 5, 10, and 15 years survival rates were 91.7%, 81.4%, and 71.3%, and the cardiac death-free survival rates were 95.8%, 91.7%, and 88.6%, respectively. The cumulative patency rate of the GEA graft was 98.5% at 1 month, 93.7% at 1 year, 86.2% at 5 years, and70.2% at 10 years, respectively. In 372 skeletonized GEA grafts with 452 distal anastomoses, thepatency rate at immediate, 1, and 5 years after surgery was 98.6%, 94.9%, and 88.4%, respectively. In conclusion the GEA graft is a safe and effective arterial conduit for CABG.

